# Sex, Lies, and Coronary Artery Disease

**DOI:** 10.3390/jcm10143114

**Published:** 2021-07-15

**Authors:** Helena Martínez-Sellés, David Martínez-Sellés, Manuel Martínez-Sellés

**Affiliations:** 1 Medical School of the Complutense, University of Madrid, 28040 Madrid, Spain; helena03@ucm.es; 2 Medical School of the Autonomous, University of Barcelona, 08193 Barcelona, Spain; David.MartinezSelles@e-campus.uab.cat; 3 Cardiology Department, Hospital General Universitario Gregorio Marañón, Calle Doctor Esquerdo, 46, 28007 Madrid, Spain

Epidemiological and clinical data have shown clear differences in several aspects of cardiovascular disease, particularly in the case of coronary artery disease (CAD), between men and women, including risk factors, response to therapy, quality of care, and natural history [1]. The influence of sex is seen on cardiovascular risk factors, mean age, presentation characteristics, diagnostic testing, treatments, and prognosis. CAD develops 7 to 10 years later in women than in men but still is the major cause of death in women. The risk of CAD in women is often underestimated due to the misperception that females are ‘protected’ against CAD and to the fact that women have been under-represented in clinical trials. Moreover, the under-recognition of heart disease and differences in clinical presentation in women lead to misdiagnosis and less aggressive treatment strategies [2]. Women have smaller vessels and sex-related differences in atherosclerotic plaque characteristics have been suggested [3], with microvascular dysfunction and diffuse CAD without obstructive lesions being more prevalent in women than in men. Women receive less evidence-based treatment than their male counterparts and CAD in females has a worse outcome than in males, when no adjustments are made for other characteristics such as age and comorbidities [4]. It seems that sex is also associated with CAD risk but sex-based differences in prognosis are largely explained by age [5], clinical differences at presentation, and the severity of angiographically documented disease [6]. Complications such as heart failure, cardiogenic shock, and re-infarction are more frequent in women than in men. Elderly women with acute coronary syndromes also present frailty and readmissions more frequently than men [7]. 

The reasons that explain the role of sex in CAD are multifactorial. Clear sexual differences in CAD genetic predisposition have been described [8], and the Y chromosome has an important role in inheritance of CAD risk and atherosclerosis [9]. Sex chromosome and hormonal mechanisms interact [10] until menopause finishes the exposure to endogenous estrogens during the fertile period that seems to delay the manifestation of atherosclerotic disease. In fact, early menopause decreases life expectancy by two years [11]. On the other hand, a harmful cardiovascular risk profile may also influence the age at menopause. Estrogens have a regulating effect not only on lipids, but also on inflammatory markers and coagulation factors. These sexual hormones also promote a direct vasodilatory effect. Pregnancy is exclusive to women and produces relevant changes in the cardiovascular system, and most are reversible, but some persist in the long term [12]. Although these changes seem to mainly have a protective effect, pregnancy-related disorders (gestational diabetes and preeclampsia) increase the risk of future CAD. For instance, women with preeclampsia have twice the risk of CAD [13]. Pregnancy is also a risk factor for spontaneous coronary artery dissection, an uncommon condition that might be a devastating event.

Women are at less risk of CAD than men, but have unique risk factors, such as being postmenopausal, oral contraceptive use, pregnancy-related disorders, and possibly preterm delivery and polycystic ovary syndrome. Other cardiovascular risk factors, such as cigarette smoking, hypertension, hyperlipidemia, and certain psychosocial factors, pose risks for both men and women, but have aspects that are unique to women. Risk factors also have a different prevalence according to sex [4]. Women with CAD are, in general, older than men, with more common traditional risk factors, except tobacco. However, smoking increases the risk of CAD relatively more in females than in men, particularly at younger ages. This could be related to the fact that, in premenopausal women, tobacco consumption causes a downregulation of estrogen-dependent vasodilatation. In addition, oral contraceptive use increases the prothrombotic effect of smoking. Central obesity and type 2 diabetes are more common in postmenopausal women than in their male counterparts [14], the risk of CAD is higher in diabetic women compared with diabetic males, and the effect of diabetes in the prognosis seems to be stronger in women [15]. Systolic blood pressure rises more steeply in aging females compared with males, and, again, this seems to be related to the decrease in estrogen levels seen in menopause. During menopause, total cholesterol and low-density lipoprotein (LDL) levels rise and, above 65 years of age, mean LDL cholesterol is higher in women compared with men. 

The clinical presentation of CAD is frequently different in women and men. In fact, what is known as ‘typical’ chest pain was described mainly in young male populations. Women are more likely to present with less well-defined symptoms or even without chest pain, have more concomitant vaso-vegetative symptoms, and tend to describe their chest pain as ‘crushing/pressure/squeezing/tightness’, whereas men mostly describe it as ‘aching/dull’. These ‘atypical’ symptoms frequently delay investigations and treatment for CAD. Traditional diagnostic methods might be less effective in women. Nonspecific ECG abnormalities are more common in women than in men. At younger ages, endogenous estrogen levels can induce ECG changes, mimicking ischemia, and women have less extensive ST-segment elevations. Breast tissue appears to have a practically negligible effect on ECG amplitudes [16] and the recommendation is to place chest electrodes on women on the breast rather than under the breast, to facilitate the precision of electrode placement at the correct horizontal level and at the correct lateral positions. However, this recommendation is frequently not followed. We should also keep in mind that ECG interpretation can be misleading in women with breast implants [17]. Women with chest pain are less likely to undergo an exercise ECG [18] and coronary angiography [19]. The interpretation of noninvasive diagnostic testing is less reliable in women compared with men, especially at young ages when the prevalence of CAD is relatively low. However, this fact does not justify the referral bias that produces a less intensive investigation in women than in their male counterparts, a bias that increases in patients ≥75 years old [20].

Regarding the therapeutic approach, there are no relevant differences in guideline-recommended treatment between men and women. However, lower rates of revascularization have been noted in women but the evidence for early invasive strategy of patients with non-ST-segment elevation acute coronary syndrome is lower in women than in men. Women with ST-segment elevation myocardial infarction experience reperfusion delays more frequently than men and are more likely to exceed reperfusion time guidelines [21]. Mortality after coronary artery bypass surgery is higher in women compared with men, particularly at younger ages. Many factors might explain this fact, and smaller vessel size is probably an important one, but comorbid conditions and age definitely have a role. Sex also influences behavioral and psychological factors. Less physical activity, cardiac rehabilitation attendance, and statin use in women than in men have been described [22]. Women with CAD, and those with nonobstructive coronary artery disease, report more psychosocial distress compared with men [23] and have a higher rate of depression following acute myocardial infarction [24]. Psychological factors might also explain, at least in part, the higher prevalence of Takotsubo syndrome in women than in men [25] and why emotional stress is more frequently found in females [26]. 

In conclusion, the supposed female protection from CAD is a lie that leads to underestimating the risk of this condition in women. CAD in women has specific characteristics but is frequently under-recognized and undertreated. A greater awareness of sex-related differences is needed to improve the management of CAD in women. The development of directed problem-solving tools and treatments is needed to close the sex gap in outcomes.
